# Polymorphism of nucleotide binding domain-like receptor protein 3 (NLRP3) increases susceptibility of total urinary arsenic to renal cell carcinoma

**DOI:** 10.1038/s41598-020-63469-8

**Published:** 2020-04-20

**Authors:** Chi-Jung Chung, Bo-Ying Bao, Ying-Chin Lin, Ya-Li Huang, Horng-Sheng Shiue, Pui-Lam Ao, Yeong-Shiau Pu, Chao-Yuan Huang, Yu-Mei Hsueh

**Affiliations:** 1 Department of Public Health, College of Public Health, China Medical University, Taichung, Taiwan; 2 Department of Medical Research, China Medical University and Hospital, Taichung, Taiwan; 3 Department of Pharmacy, College of Pharmacy, China Medical University, Taichung, Taiwan; 4 Sex Hormone Research Center, China Medical University Hospital, Taichung, Taiwan; 5 Department of Nursing, Asia University, Taichung, Taiwan; 6 Department of Family Medicine, Wan Fang Hospital, Taipei Medical University, Taipei City, Taiwan; 7 Department of Family Medicine, School of Medicine, College of Medicine, Taipei Medical University, Taipei, Taiwan; 8 Department of Geriatric Medicine, School of Medicine, College of Medicine, Taipei Medical University, Taipei, Taiwan; 9 Department of Public Health, School of Medicine, College of Medicine, Taipei Medical University, Taipei, Taiwan; 10 Department of Chinese Medicine, College of Medicine, Chang Gung University Taoyuan, Taoyuan, Taiwan; 11 School of Public Health, College of Public Health, Taipei Medical University, Taipei, Taiwan; 12 Department of Urology, National Taiwan University Hospital, College of Medicine National Taiwan University, Taipei, Taiwan

**Keywords:** Environmental impact, Cancer epidemiology

## Abstract

Our study showed that total urinary arsenic concentrations were positively correlated with renal cell carcinoma (RCC). Chronic inflammation is a key player in the development of RCC. This study explored the association between nucleotide-binding domain-like receptor protein 3 (*NLRP3*) genotypes and the development of RCC. We also investigated whether any of the *NLRP3* genotypes modified the risk between arsenic and RCC. We recruited 350 RCC patients and 700 age-sex matched controls. RCC was confirmed by pathological assessment following surgical resection or image-guided biopsy of a renal tumor. Fifteen sites of N*LRP3* gene polymorphisms were identified using the Agena Bioscience MassARRAY platform. The concentrations of the urinary arsenic species were determined by HPLC-HG-AAS. There was a significant dose-dependent association between arsenic and RCC. In addition, six of thirteen *NLRP3* alleles, including rs12239046 C, rs10925025 G, rs1539019 C, rs10925026 A, rs10157379 T, and rs12143966 A, had increased odds ratios (ORs) for RCC than other *NLRP3* alleles. Among these sites, we found the novel haplotype of five tag-SNPs (C-A-A-A-A) was significantly related to RCC, the OR and 95% confidence interval was 1.44 (1.08–1.92). Furthermore, participants with high total urinary arsenic levels and the *NLRP3* rs1539019 C allele had significantly multiplicative and additive interactions for the risk of RCC (p _interaction_ = 0.012). This study is the first to identify the modified effects of *NLRP3* risk alleles involved in the association between arsenic and RCC risk in a population with low arsenic exposure.

## Introduction

Renal cell carcinoma (RCC) represents the most deadly urological malignancy and accounts for 2 to 3% of all adult malignancies. RCC is most commonly diagnosed between the ages of 50 and 75 years old with a ratio of males to females of 1.5:1^1^. The incidence of RCC in most countries has been increasing over the past decade^2^. In Taiwan, the incidence trend and average annual percentage increase for kidney cancer from 2002 to 2012 was 5.1 and 2.9% for men and women, respectively^3^. Although cigarette smoking, obesity, and hypertension have been identified as risk factors for RCC^4^, the etiology of RCC is still unclear.

Chronic inflammation is a key player in the occurrence and development of RCC^5^. It can be caused by environmental exposure, obesity, tumorigenic pathogens, immune deregulation, and autoimmunity^6^. Chronic inflammation promotes tumorigenesis by enhancing genomic instability, inducing oncogenic mutations, and altering the immune response^6^. In addition, arsenic is a Group I carcinogen has been shown to cause lung, skin, liver, kidney and bladder cancer^7^. Our study showed that subjects with a high total arsenic concentration in their urine had a high odds ratio (OR) for RCC^8^, even if they are exposed to low arsenic levels. Several studies suggested that arsenic-induced nephrotoxicity may be through activation of inflammasomes and induction of cyclooxygenase-2 (Cox-2) as well as COX-2-derived prostanoids upregulation^9–11^. Therefore, whether arsenic induces RCC through an inflammatory response is a topic worthy of discussion.

Inflammasomes are newly discovered immune complexes that help a host defend against physiological aberrations and infectious agents^12^. Absent in melanoma 2 (AIM2)-like receptors (ALRs), leucine-rich repeat-containing receptor (NLR), or the nucleotide-binding domain initiate the inflammasome complex. Nucleotide-binding domain-like receptor protein 3 (NLRP3), caspase-1, and adaptor ASC (apoptosis-associated spot-like protein) constitute the NLRP3 inflammasome, which can be activated through *in vivo* cell damage and death or invasion of foreign pathogens^13^. One recent study reported that arsenic could activate the NLRP3 inflammasome and induce pyroptosis^14^. However, mercury and arsenic can inhibit interleukin (IL)-1β and IL-18 secretion, which is caused by the activation of both the classical and non-classical NLRP3 inflammasomes in macrophages, suggesting that exposure to these heavy metals could destroy the inflammasome-mediated immune responses and cause unexpected side effects^15^. Because of the inconsistency of these findings, the association between NLRP3 inflammasomes and arsenic exposure remains unclear. In addition, a study has found that NLRP3 is overexpressed in patients with bladder cancer^16^. However, the association between NLRP3 and RCC needs investigation.

NLRP3 gene variants may influence NLRP3 mRNA stability and expression^17^. Recent studies have identified an association between *NLRP3* gene polymorphisms and both increased blood pressure^18^ and coronary artery disease^19^. However, no studies have examined their association with the development of RCC. Therefore, the aims of the study were to investigate the relationship between *NLRP3* genotypes and the risk of RCC and explore whether *NLRP3* gene polymorphisms could modify the risk between arsenic and RCC.

## Materials and Methods

### Study subjects

This study was a case-control study. To prevent age and gender from confounding the risk of RCC, we matched the age and gender of the control group with those of the case group. Pathologically-confirmed RCC patients (350) and 700 age- and gender-matched controls (i.e., without RCC or any other malignancy) were recruited from our past study^20^. Among the RCC patients, about 70% had grade II or III tumors, including 262 clear-cell, 25 papillary, 21 chromophobe, one collecting duct, one sarcomatoid, and five “other” cases. No information was available for 35 of the cases. The Research Ethics Committee of National Taiwan University Hospital approved the study, which complied with the World Medical Association Declaration of Helsinki. All study subjects provided their informed consent before specimen and data collection.

All study subjects were Taipei residents and drank tap water with arsenic levels within the World Health Organization standards^21^. Although Taipei does not have an arsenic-related factory, the urinary arsenic species present in the study subjects may be due to exposure from seafood^22^, cereals^23^, edible oil^24^, and agricultural rice^25^.

### Biological specimen collection and questionnaire interview

The questionnaire interview, the content of the questionnaires, and the methods used for collecting blood and urine samples were previously described^8^. Peripheral blood samples (5 to 8 mL) were collected using EDTA-vacuum syringes. The buffy coat was separated for DNA extraction and gene polymorphism determination. Spot urine can reflect arsenic excretion concentrations over 24 h^26^. Total urinary arsenic levels were adjusted by the urinary creatinine concentration for variation in the hydration states^27^.

### Measurement of arsenic species in urine

The methods for measuring arsenite (As^III^), dimethylarsenic acid (DMA^V^), monomethylarsonic acid (MMA^V^), and arsenate (As^V^) in urine were previously described^28^. Urine sample pretreatment, the method of measurement and validity, and the reliability of the arsenic species in the urine are described in Supplemental Table S1. The intake of fish, shellfish, or any other seafood did not affect the method of determining the arsenic species^29^.

### Genetic polymorphisms determination

**Figure 1:**
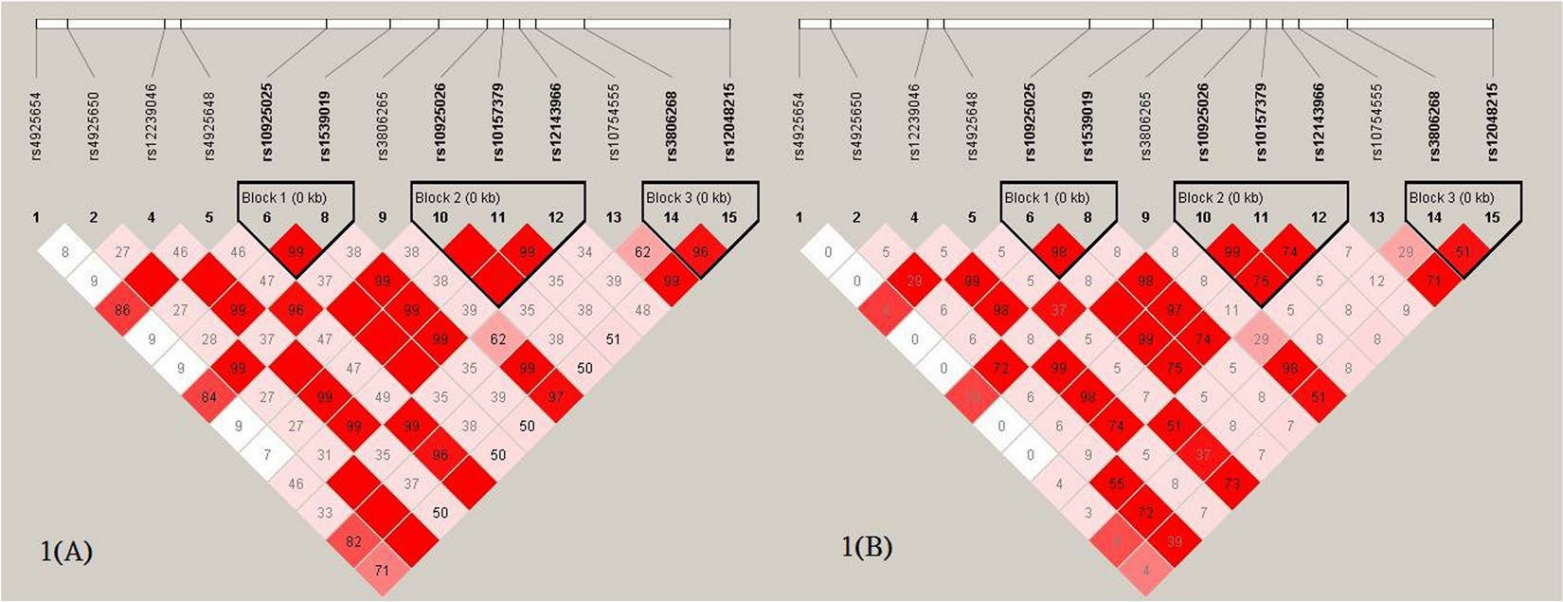
(**A**) Lewontin’s D’ of the *NLRP3* block 1 (*NLRP3* rs10925025 and *NLRP3* rs1539019), *NLRP3* block 2 (*NLRP3* rs10925026, *NLRP3* rs10157379, and *NLRP3* rs12143966), and *NLRP3* block 3 (*NLRP3* rs3806268 and *NLRP3* rs12048215) polymorphisms. (**B**) *r*^2^ values for each pair of polymorphisms of NLRP3 block 1, NLRP3 block 2, and NLRP3 block 3.

DNA extraction was performed using proteinase K digestion and phenol and chloroform extraction. Seventeen common single nucleotide polymorphisms (SNPs) in the *NLRP3* gene region were initially selected from the Han Chinese in Beijing HapMap data with a minor-allele frequency of ≥ 0.2. However, two SNPs failed during the genotyping assay design. Genotyping of 15 SNPs was performed using the Agena Bioscience MassARRAY iPLEX system, according to manufacturer’s instructions (National Genome Medicine Center, Taipei, Taiwan). Two SNPs did not fit the Hardy-Weinberg equilibrium and were removed. Therefore, 13 *NLRP3* SNPs were included in the analyses. The *NLRP3* gene exhibited three haplotype blocks as shown in Fig. 1.

### Statistical analysis

The values for the As^III^ and As^V^ (InAs), MMA^V^, and DMA^V^ species in the urine were added to determine the total urinary arsenic concentration. The differences in the continuous variables between two groups were compared using the Student’s t-test. Multiple logistic regression models were used to calculate the OR and 95% confidence interval (CI). The continuous variable of the total urinary arsenic levels in the controls was categorized, and the resulting tertile was defined as the cutoff point. The linear trends for the ORs across the strata of independent variables were tested by categorizing the independent variables and treating the score variables as continuous. Haploview 4.1 software was used to calculate D’ and r^2^ of the Lewontin to determine the strength of the linkage disequilibrium (LD) intensity^30^. This cutoff value represents the median of the total urinary arsenic concentration of the controls (15.6 μg/g creatinine) and was used for the interaction analysis. We tested the multiplicative interaction of the total urinary arsenic concentration and each *NLRP3* genotype using a product term in the logistic regression model. Additive interactions were evaluated using the Synergy (S) Index^31^. The analysis of all data used SAS 9.4 software (Cary, NC, USA). A two-sided 0.05 < *p* < 0.1 and *p* < 0.05 was considered marginally significant and statistically significant, respectively. The statistical power of this study was calculated using Power and Sample Size Calculation online software (http://sampsize.sourceforge.net/iface/s3.html). Based on the number of samples in this study, the odds ratio was about 2, the exposed controls were 5%, the alpha risk was 5%, and the controls/case ratio was 2, resulting in a power of about 80%.

## Results

### Sociodemographic characteristics of RCC cases and controls

The mean age of 350 RCC patients and 700 healthy controls were 59.29 ± 0.70 and 60.12 ± 0.49 years, respectively in this study. Subjects with higher educational level or who were alcohol drinkers had a lower OR for RCC than those with a lower level of education or who were non-drinkers. Cigarette smoking ≥ 21 pack-years significantly increased the OR 1.60-fold for RCC compared to non-smokers (Table 1). We also analyzed the consumption effect of cumulative cigarette smoking with all 13 *NLRP3* SNPs with genotypic form. However, we did not find any difference in the consumption of cumulative cigarette smoking for different genotypes of any *NLRP3* SNPs (Supplemental Table S2). Hypertension and diabetes were significantly associated with RCC with ORs (95% CI) of 2.76 (2.07–3.67) and 2.65 (1.80–3.90), respectively (data not shown). In this study, total urinary arsenic levels of RCC cases and controls were 23.72 ± 1.19 and 18.91 ± 0.50 μg/g creatinine, but in the arseniasis endemic area of Taiwan, those of urothelial carcinoma cases and controls were 69.6 ± 11.4 and 63.7 ± 14.2 μg/L respectively^32^.

**Table 1:** Sociodemographic characteristics, lifestyle, disease histories, urinary creatinine, and urinary total arsenic levels of RCC cases and non-RCC controls.

	RCC Cases (n = 350) N (%)	Controls (n = 700) N (%)
Age (years) (Mean ± SD)	59.29 ± 0.70	60.12 ± 0.49
Gender		
Male	231 (66.00)	462 (66.00)
Female	119 (34.00)	238 (34.00)
Education		
Illiterate/Elementary school	78 (22.29)	131 (18.71)
Junior/Senior high school	143 (40.86)	240 (34.29)
College or above	129 (36.86)	329 (47.00)
Smoking		
No	221 (63.32)	472 (67.43)
Former or current	128 (36.68)	228 (32.57)
Cumulative cigarette smoking (pack-years) (Mean ± SD)	10.73 ± 1.11	8.34 ± 0.70
0	221 (65.38)	472 (69.01)
<21	44 (13.02)	106 (15.50)
≥21	73 (21.60)	106 (15.50)
Alcohol consumption		
No	274 (78.29)	418 (59.71)
Occasional or frequent	76 (21.71)	282 (40.29)
Diabetes		
No	284 (81.38)	641 (91.57)
Yes	65 (18.62)	59 (8.43)
Hypertension		
No	188 (53.71)	516 (73.71)
Yes	162 (46.29)	184 (26.29)
Urinary creatinine (mg/dL) (Mean ± SD)	77.21 ± 2.80^a^	133.46 ± 3.36^a^
Total urinary arsenic (μg/L) (Mean ± SD)	18.01 ± 1.11^a^	22.79 ± 0.72^a^
<12.2	173 (33.43)	234 (33.43)
12.2–26.2	100 (28.57)	233 (33.29)
≥26.2	77 (22.00)	233 (33.29)
Total urinary arsenic (μg/g creatinine) (Mean ± SD)	23.72 ± 1.19^a^	18.91 ± 0.50^a^
<11.5	90 (25.71)	234 (33.43)
11.5–20.4	107 (30.57)	233 (33.29)
≥ 20.4	153 (43.71)	233 (33.29)

SD: standard deviation. RCC: renal cell carcinoma.

^§^*P* < 0.05 for the trend test; ^a^p < 0.05 calculated using the Wilcoxon rank–sum test.

### Alleles, genotypes, and haplotype of NLRP3 gene and RCC risk

A marginally significant increased RCC risk was showed among participants with the *NLRP3* rs12239046 T allele compared to the *NLRP3* rs12239046 C allele, the OR (95% CI) was 1.20 (0.98 to 1.47). Participants with the *NLRP3* rs10925025 (G vs. A allele), *NLRP3* rs1539019 (C vs. A allele), *NLRP3* rs10925026 (A vs. C allele), *NLRP3* rs10157379 (T vs. C allele), and *NLRP3* rs12143966 (A vs. G allele) genotypes had similar 1.20–1.22-fold risks to that of N*LRP3* rs12239046 (C vs. T allele). However, for the *NLRP3* rs10925025 GG vs. AA genotype, the OR (95% CI) of RCC was 1.43 (0.95–2.17); for the *NLRP3* rs10925026 AA vs. CC genotype, the OR (95% CI) of RCC was 1.42 (0.94–2.15); for the *NLRP3* rs1539019 AA vs. CC genotype, the OR (95% CI) of RCC was 0.70 (0.46–1.05). These comparisons were all marginally significant. For the *NLRP3* rs12143966 AA genotype compared to the GG genotype, the OR (95% CI) of RCC was 1.50 (1.01–2.22). Other *NLRP3* genotypes were not associated with RCC risk (Table 2).

**Table 2:** Alleles, genotype and haplotypes of inflammasome gene and the risk of RCC.

Alleles and haplotypes of *NLRP3*	RCC Cases (n = 350)	Controls (n = 700)	Crude ORs (95% CI)	Multivariate adjusted ORs (95% CI)^a^
**SNP1: rs4925654**				
G	589 (84.63)	1192 (85.26)	1.00	1.00
A	107 (15.37)	206 (14.74)	1.05 (0.82–1.36)	1.12 (0.85–1.47)
GG	250 (71.84)	509 (72.82)	1.00	1.00
GA	89 (25.57)	174 (24.89)	1.04 (0.78–1.40)	1.09 (0.79–1.51)
AA	9 (2.59)	16 (2.29)	1.17 (0.51–2.68)	1.36 (0.56–3.30)
**SNP2: rs4925650**				
G	357 (51.00)	761 (54.36)	1.00	1.00
A	343 (49.00)	639 (45.64)	1.14 (0.95–1.37)	1.12 (0.92–1.37)
GG	97 (27.71)	203 (29.00)	1.00	1.00
GA	163 (46.57)	355 (50.71)	0.97 (0.71–1.31)	1.00 (0.71–1.39)
AA	90 (25.71)	142 (20.29)	1.34 (0.93–1.91)	1.27 (0.86–1.87)
**SNP3: rs12239046**				
T	267 (38.25)	590 (42.14)	1.00	1.00
C	431 (61.75)	810 (57.86)	1.18 (0.98–1.42)^+^	1.20 (0.98–1.47)^+^
CC	136 (38.97)	236 (33.71)	1.00	1.00
CT	159 (45.56)	338 (48.29)	0.82 (0.62–1.09)	0.84 (0.62–1.14)
TT	54 (15.47)	126 (18.00)	0.74 (0.51–1.09)	0.71 (0.47–1.07)
**SNP4: rs4925648**				
T	169 (24.14)	353 (25.21)	1.00	1.00
C	531 (75.86)	1047 (74.79)	1.06 (0.86–1.31)	1.04 (0.83–1.30)
CC	203 (58.00)	398 (56.86)	1.00	1.00
CT	125 (35.71)	251 (35.86)	0.97 (0.74–1.28)	1.03 (0.77–1.39)
TT	22 (6.29)	51 (7.29)	0.83 (0.49–1.41)	0.83 (0.46–1.47)
**SNP5: rs10925025**				
A	267 (38.25)	588 (42.30)	1.00	1.00
G	431 (61.75)	802 (57.70)	1.18 (0.98–1.43)^+^	1.20 (0.98–1.47)^+^
AA	53 (15.19)	125 (17.99)	1.00	1.00
AG	161 (46.13)	338 (48.63)	1.13 (0.78–1.65)	1.21 (0.81–1.81)
GG	135 (38.68)	232 (33.38)	1.37 (0.93–2.02)	1.43 (0.95–2.17)^+^
**SNP6: rs1539019**				
A	265 (37.86)	585 (41.91)	1.00	1.00
C	435 (62.14)	811 (58.09)	1.18 (0.98–1.43)^+^	1.21 (0.99–1.48)^+^
CC	138 (39.43)	237 (33.95)	1.00	1.00
CA	159 (45.43)	337 (48.28)	0.82 (0.62–1.08)	0.83 (0.61–1.13)
AA	53 (15.14)	124 (17.77)	0.73 (0.50–1.08)	0.70 (0.46–1.05)^+^
**SNP7: rs3806265**				
C	300 (42.86)	653 (46.64)	1.00	1.00
T	400 (57.14)	747 (53.36)	1.17 (0.97–1.40)	1.15 (0.94–1.40)
TT	112 (32.00)	200 (28.57)	1.00	1.00
TC	176 (50.29)	347 (49.57)	0.90 (0.67–1.21)	0.92 (0.67–1.27)
CC	62 (17.71)	153 (21.86)	0.72 (0.49–1.04) +	0.74 (0.49–1.11)
**SNP8: rs10925026**				
C	267 (38.14)	587 (42.05)	1.00	1.00
A	433 (61.86)	809 (57.95)	1.18 (0.98–1.42)^+^	1.20 (0.98–1.47)^+^
CC	53 (15.14)	125 (17.91)	1.00	1.00
CA	161 (46.00)	337 (48.28)	1.14 (0.78–1.65)	1.21 (0.81–1.81)
AA	136 (38.86)	236 (33.81)	1.36 (0.93–2.00)	1.42 (0.94–2.15)^+^
**SNP9: rs10157379**				
C	268 (38.29)	589 (42.19)	1.00	1.00
T	432 (61.71)	807 (57.81)	1.18 (0.98–1.42)	1.20 (0.98–1.46)^+^
CC	53 (15.14)	124 (17.77)	1.00	1.00
CT	162 (46.29)	341 (48.85)	1.12 (0.77–1.63)	1.20 (0.80–1.79)
TT	135 (38.57)	233 (33.38)	1.36 (0.92–2.00)	1.42 (0.94–2.14)
**SNP10: rs12143966**				
G	307 (44.62)	679 (49.20)	1.00	1.00
A	381 (55.38)	701 (50.80)	1.20 (1.00–1.44)^+^	1.22 (1.00–1.49)^+^
GG	70 (20.35)	175 (25.36)	1.00	1.00
GA	167 (48.55)	329 (47.68)	1.27 (0.91–1.77)	1.39 (0.97–2.00)^+^
AA	107 (31.10)	186 (26.96)	1.43 (0.99–2.06) ^+^	1.50 (1.01–2.22)
**SNP11: rs10754555**				
G	259 (37.00)	552 (39.43)	1.00	1.00
C	441 (63.00)	848 (60.57)	1.11 (0.92–1.34)	1.10 (0.89–1.34)
CC	147 (42.00)	261 (37.29)	1.00	1.00
CG	147 (42.00)	326 (46.57)	0.80 (0.61–1.06)	0.84 (0.62–1.13)
GG	56 (16.00)	113 (16.14)	0.87 (0.60–1.27)	0.89 (0.59–1.34)
**SNP12: rs3806268**				
G	298 (42.57)	651 (46.50)	1.00	1.00
A	402 (57.43)	749 (53.50)	1.17 (0.98–1.41)^+^	1.15 (0.94–1.40)
AA	113 (32.29)	203 (29.00)	1.00	1.00
AG	176 (50.29)	343 (49.00)	0.92 (0.68–1.23)	0.95 (0.69–1.30)
GG	61 (17.43)	154 (22.00)	0.70 (0.48–1.03)	0.74 (0.49–1.11)
**SNP13: rs12048215**				
G	207 (29.57)	447 (31.93)	1.00	1.00
A	493 (70.43)	953 (68.07)	1.12 (0.92–1.36)	1.98 (0.88–1.35)
AA	176 (50.29)	331 (47.29)	1.00	1.00
AG	141 (40.29)	291 (41.57)	0.91 (0.70–1.20)	0.95 (0.71–1.28)
GG	33 (9.43)	78 (11.14)	0.78 (0.50–1.23)	0.81 (0.50–1.31)
***NLRP3*** **block 1:** ***NLRP3*** **rs10925025 and** ***NLRP3*** **rs1539019**				
A-A	265 (37.86)	585 (41.79)	0.85 (0.70–1.02)	0.83 (0.68–1.02)^+^
A-C	2 (0.29)	4 (0.29)	0.93 (0.17–5.12)	1.21 (0.21–7.09)
G-A	0	2 (0.14)		
G-C	433 (61.86)	809 (57.79)	1.00	1.00
***NLRP3*** **block 2:** ***NLRP3*** **rs10925026**, ***NLRP3*** **rs10157379, and** ***NLRP3*** **rs12143966**				
A-C-A	1 (0.14)	3 (0.21)	0.61 (0.06–5.87)	0.76 (0.07–8.30)
A-C-G	0	1 (0.07)		
A-T-A	387 (55.29)	706 (50.50)	1.00	1.00
A-T-G	45 (6.43)	101 (7.22)	0.81 (0.56–1.18)	0.80 (0.54–1.20)
C-C-G	267 (38.14)	587 (41.99)	0.83 (0.69–1.00)^+^	0.82 (0.66–1.00)^+^
***NLRP3*** **block 3:** ***NLRP3*** **rs3806268 and** ***NLRP3*** **rs12048215**				
A-A	397 (56.71)	743 (53.07)	1.00	1.00
A-G	5 (0.71)	6 (0.43)	1.56 (0.47–5.14)	2.09 (0.55–7.91)
G-A	96 (13.71)	210 (15.00)	0.86 (0.65–1.12)	0.88 (0.65–1.17)
G-G	202 (28.86)	441 (31.50)	0.86 (0.70–1.05)	0.88 (0.70–1.10)
***NLRP3*** **Five Tag-SNPs**				
A-A-A-G-A	0	1 (0.07)		
A-A-G-G-A	0	1 (0.07)		
A-C-G-A-A	211 (30.14)	448 (32.00)	1.00	1.00
A-C-G-A-G	4 (0.57)	6 (0.43)	1.42 (0.40–5.07)	1.74 (0.41–7.38)
A-C-G-G-A	31 (4.43)	65 (4.64)	1.01 (0.64–1.60)	0.96 (0.58–1.57)
A-C-G-G-G	19 (2.71)	66 (4.71)	0.61 (0.36–1.05)^+^	0.64 (0.36–1.13)
C-A-A-A-A	149 (21.29)	220 (15.71)	1.44 (1.10–1.87)	1.44 (1.08–1.92)*
C-A-A-A-G	1 (0.14)	0		
C-A-A-G-A	64 (3.05)	139 (9.93)	0.98 (0.70–1.37)	1.04 (0.72–1.50)
C-A-A-G-G	174 (24.86)	350 (25.00)	1.06 (0.83–1.35)	1.07 (0.82–1.40)
C-A-G-A-A	37 (5.29)	75 (5.36)	1.05 (0.68–1.60)	1.05 (0.66–1.65)
C-A-G-G-G	8 (1.14)	25 (1.79)	0.68 (0.30–1.53)	0.71 (0.29–1.76)
C-C-G-G-A	1 (0.14)	4 (0.29)	0.53 (0.06–4.78)	0.64 (0.07–6.25)
C-C-G-G-G	1 (0.14)	0		
C-A-A-A-A	149 (21.29)	220 (15.71)	1.45 (1.15–1.83)	1.43 (1.11–1.84)*
others	551 (78.71)	1180 (84.29)	1.00	1.00

^+^0.05 ≤ P < 0.1 and *P < 0.05.

^a^Model was adjusted by age, sex, education, cumulative cigarette smoking, alcohol consumption, diabetes, and hypertension.

*NLRP3* Five Tag-SNPs: rs1539019, rs10925026, rs12143966, rs3806268, and rs12048215.

**Figure 2:**
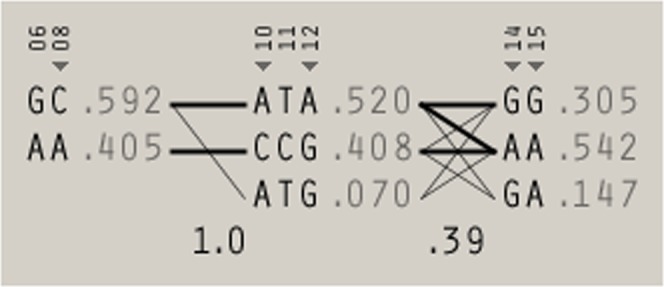
Five Tag-SNPs of *NLRP3* gene.

Lewontin’s D’ of polymorphisms *NLRP3* block 1 (*NLRP3* rs10925025 and *NLRP3* rs1539019), *NLRP3* block 2 (*NLRP3* rs10925026, *NLRP3* rs10157379, and *NLRP3* rs12143966), and *NLRP3* block 3 (*NLRP3* rs3806268 and *NLRP3* rs12048215) ranged from 0.96 to 0.99 indicating the LD (Fig. 1A), and *r*^2^ values (Fig. 1B) for each pair of polymorphisms. The A-A haplotype of *NLRP3* block 1 had a marginally significantly inverse OR for RCC compared to the G-C haplotype. Similarly, The OR for RCC was marginally significantly lower in the C-C-G haplotype of *NLRP3* block 2 than that of the A-T-A haplotype. Further we found out five Tag-SNPs of *NLRP3* gene from above seven sites of SNP, including rs1539019, rs10925026, rs12143966, rs3806268, and rs12048215 (Fig. 2). The results showed the C-A-A-A-A haplotype of the *NLRP3* Five Tag-SNPs had a significantly higher risk of RCC than other haplotypes, the OR was 1.43 (95% CI = 1.11 to 1.84).

We analyzed whether there were differences in the total urinary arsenic concentrations of the different genotypes of the 13 *NLRP3* genes. We found that the total urinary arsenic concentration of the *NLRP3* rs4925650 GA genotype was significantly higher than that of the GG genotype in all study subjects. However, the total urinary arsenic concentration of the *NLRP3* rs4925650 AA genotype was significantly higher than those of the GA genotype in RCC patients. The total urinary arsenic concentration of the *NLRP3* rs3806265 TT genotype was significantly higher than that of the CC genotype in all study subjects. In addition, the total urinary arsenic concentration of the *NLRP3* rs3806268 AA genotype was significantly higher than that of the GG genotype in all study subjects (Supplemental Table S3). However, these *NLRP3* SNPs were not associated with the risk of RCC.

### Joint effects of total urinary arsenic levels and *NLRP3* polymorphisms on RCC risk

For the *NLRP3* rs1539019 C allele and a total urinary arsenic concentration ≥ 15.6 μg/g creatinine, the risk of developing RCC increased with exposure to an increasing number of risk factors (i.e., none, one, or both risk factors). Subjects with the *NLRP3* rs1539019 C allele and total urinary arsenic concentration of ≥ 15.6 μg/g creatinine had a higher OR of RCC (2.33, 1.70–3.19) compared to those with the *NLRP3* rs1539019 A allele, whose total urinary arsenic concentration was <15.6 μg/g creatinine after multivariable adjustment (Table 3). The *NLRP3* rs1539019 C allele tended to multiplicatively and additively interact significantly with high total urinary arsenic concentrations to change the OR of RCC. In addition, the *NLRP3* rs12239046 C, *NLRP3* rs10925025 G, *NLRP3* rs10925026 A, and *NLRP3* rs10157379 T alleles tended to multiplicatively interact with high total urinary arsenic levels to affect RCC risk. However, the *NLRP3* rs12143966 A allele and *NLRP3* Five Tag-SNPs C-A-A-A-A haplotype did not interact with a high total urinary arsenic concentration in the risk of RCC.

**Table 3:** The interaction of total urinary arsenic level and inflammasome gene polymorphisms on RCC risk.

Total arsenic^b^	Polymorphisms of *NLRP3*	Case/Control Number	Multivariate ORs^a^ (95% CI)	P_interaction_	S index
Total arsenic	rs12239046			0.0219	0.66 (0.36–1.23)
<15.6	T	93/303	1.00^§^		
<15.6	C	179/397	1.60 (1.17–2.20)		
≥ 15.6	T	174/287	2.30 (1.65–3.20)		
≥ 15.6	C	252/413	2.26 (1.66–3.09)		
Total arsenic	rs10925025			0.0172	0.66 (0.43–1.01)
<15.6	A	92/302	1.00^§^		
<15.6	G	180/394	1.63 (1.19–2.25)		
≥ 15.6	A	175/286	2.35 (1.68–3.28)		
≥ 15.6	G	251/408	2.31 (1.69–3.17)		
Total arsenic	rs1539019			0.0116	0.65 (0.43–0.98)
<15.6	A	91/302	1.00^§^		
<15.6	C	181/396	1.67 (1.21–2.29)		
≥ 15.6	A	174/283	2.39 (1.71–3.33)		
≥ 15.6	C	254/415	2.33 (1.70–3.19)		
Total arsenic	rs10925026			0.0187	0.66 (0.43–1.01)
<15.6	C	92/301	1.00^§^		
<15.6	A	180/397	1.62 (1.18–2.23)		
≥ 15.6	C	175/286	2.33 (1.67–3.24)		
≥ 15.6	A	253/412	2.29 (1.67–3.13)		
Total arsenic	rs10157379			0.0250	0.67 (0.44–1.04)
<15.6	C	93/301	1.00^§^		
<15.6	T	179/395	1.59 (1.16–2.19)		
≥ 15.6	C	175/288	2.28 (1.64–3.17)		
≥ 15.6	T	253/412	2.26 (1.65–3.08)		
Total arsenic	rs12143966			0.0721	0.74 (0.46–1.18)
<15.6	G	109/342	1.00^§^		
<15.6	A	161/344	1.54 (1.13–2.10)		
≥ 15.6	G	198/337	2.07 (1.52–2.81)		
≥ 15.6	A	220/357	2.19 (1.61–2.96)		
Total arsenic	*NLRP3* haplotype			0.5812	1.02 (0.50–2.09)
<15.6	others	214/600	1.00^§^		
<15.6	C-A-A-A-A	58/100	1.54 (1.04–2.28)		
≥ 15.6	others	337/580	1.74 (1.38–2.19)		
≥ 15.6	C-A-A-A-A	91/120	2.31 (1.64–3.26)		

^§t^Rend p-value <0.05. ^a^Model was adjusted by age, sex, cumulative cigarette smoking, alcohol consumption, diabetes, and hypertension. ^b^The units of total arsenic in urine were μg/g creatinine.

We also reanalyzed the effect of gene-gene interactions on the risk of developing RCC. The marginally significant genotypes are presented in Table 2. We found that *NLRP3* rs10925025 and *NLRP3* rs12143966 or *NLRP3* rs10925026 and *NLRP3* rs12143966 had significantly additive and multiplicative interactions on the risk of RCC (Supplemental Table S4).

## Discussion

In this study, we observed a dose-dependent relationship between total urinary arsenic levels and the OR for RCC after multivariate adjustments (i.e., the higher the total urinary arsenic, the higher the OR), which reflects the results of our previous study^8^. We found that the *NLRP3* rs12239046, *NLRP3* rs10925025, *NLRP3* rs1539019, *NLRP3* rs10925026, *NLRP3* rs10157379, and *NLRP3* rs12143966 genotypes were marginally significantly correlated with the risk of RCC. Additionally, the *NLRP3* rs1539019 C, *NLRP3* rs12239046 C, *NLRP3* rs10925025 G, *NLRP3* rs10925026 A, and *NLRP3* rs10157379 T alleles tended to multiplicatively interact with high total urinary arsenic concentrations on the risk of RCC. Specifically, the *NLRP3* rs1539019 C allele significantly additively interacted with high total arsenic concentration to change the risk of RCC.

Diabetes and hypertension were important risk factors for RCC in this study. Capitanio *et al*.^33^ also concluded in a recent review that hypertension is a critical risk factor for RCC. This risk may be caused by chronic renal hypoxia due to oxidative damage and lipid peroxidation caused by hypertension^34^. A case-control study from Sri Lanka also showed that diabetes was significantly associated with RCC^35^. This association may be because a high insulin concentration increases the concentration of insulin-like growth factor 1, which, in turn, can upregulate vascular endothelial growth factor secretion and induce tumor angiogenesis, leading to tumorigenesis and metastasis^36^. These associations need to be explored further.

RCC is a disease that involves complicated interactions between various environmental^33^ and genetic factors^37,38^. Recent study pointed out that tumor-associated immune cells play an important role in the initiation and progression of RCC^39^. The NLRP3 inflammasome is important for innate immune responses. NLRs affect the pathogenesis of many diseases, including neurodegenerative, metabolic, cardiovascular, and kidney diseases^40^. Cellular stress and tissue damage can activate NLR. One of the models of NLRP3 inflammasome activation is the dependence on reactive oxygen species^41^. Inflammasome disorders are associated with some inflammatory diseases. NLRP3 interacts with insulin resistance-associated thioredoxin-interacting protein (TXNIP). In response to reactive oxygen species, TXNIP dissociates from thioredoxin and binds to NLRP3 to activate the inflammasome. Lack of TXNIP can impair the activation of the NLRP3 inflammasome and subsequent secretion of IL-1β, which may be related to the pathogenesis of diabetes^41^. Diabetes is one of the risk factors for RCC^42^ . The association of NLRP3 with RCC and diabetes needs further investigation.

One study has shown that arsenic trioxide (As_2_O_3_) could induce nonalcoholic steatohepatitis, increase autophagy, NLRP3 inflammasome activation, and lipid accumulation, which leads to lipid-related gene dysregulation^14^. Another study demonstrated that arsenic enhanced the AIM2 inflammasome activation to increase IL-1 β/IL-18 production^11^. In contrast, Ahn *et al*.^15^ demonstrated that arsenic could inhibit the activation of the NLRP3 inflammasome in macrophages in response to lipopolysaccharide treatment, which attenuated the elevation of serum IL-1β in mice. Animal and cell culture studies showed that arsenic trioxide and other arsenic compounds inhibited NLRP3 inflammasome, caspase-1, and IL-1β inflammatory signaling, and played a major role in its anti-cancer effects^43^. Overall, the correlation between arsenic and the NLRP3 inflammasome remains unclear.

The NLRP3 inflammasome functions in the host immune response but also plays a role in the susceptibility to inflammatory disorders^44^. The *NLRP3* gene is located on the long arm of chromosome 1q44^17^. It has nine exons within its 32.9 kb sequence^17^. Paramel *et al*.^45^ demonstrated a relationship between *NLRP3* SNPs and the susceptibility to some inflammatory diseases. Approximately 60 SNPs have been identified within the *NLRP3* gene^46^. Many studies have examined the association between the *NLRP3* genotypes and cardiovascular disease. However, the results are inconsistent. The *NLRP3* rs7512998 C allele, but not the TT-genotype, has been significantly associated with higher systolic and diastolic blood pressure^18^. The *NLRP3* rs4612666 gene polymorphism may affect the risk of having a large artery atherosclerosis-induced ischemic stroke^47^. However, *NLRP3* rs12239046 allele was not associated with cardiovascular disease^48^. Fewer studies have examined the association of the *NLRP3* genotypes with cancer. One study found that the *NLRP3* rs35829419 C > A (Q705K) genotype was associated with a lower survival rate in patients diagnosed with invasive colorectal cancer^49^. However, this genotype was not associated with myeloid leukemia^50^ or pancreatic cancer^51^. However, the *NLRP3* alleles of rs10925025 G, rs1539019 C, rs10925026 A, and rs12143966 A tended to correlate with the risk of RCC in this study.

Studies exploring *NLRP3* rs1539019 genotypes showed relationships between the *NLRP3* rs1539019 TT genotype and increased risk of pneumoconiosis^52^ and *NLRP3* rs1539019 polymorphisms and heart disease^53^ but not major blunt trauma^17^. Our study is the first to demonstrate that the *NLRP3* rs1539019 C allele is marginally associated with RCC, but the five *NLRP3-*tag SNPs were found to be in LD. Haplotype analysis of these five *NLRP3* SNPs showed that haplotype C-A-A-A-A (*NLRP3* rs1539019, *NLRP3* rs10925026, *NLRP3* rs12143966, *NLRP3* rs3806268, and *NLRP3* rs12048215) significantly increased the risk of RCC compared to the other haplotypes after multivariate adjustment. Furthermore, the *NLRP3* rs1539019 C allele additively and multiplicatively interacted significantly with the total urinary arsenic concentration in the increased risk of RCC. The relationships identified in this study may result from the effect of arsenic on NLRP3 inflammasome activation, which alters caspase-1 and IL-1β levels^11^ and the risk of developing RCC. Perhaps, *NLRP3* rs1539019 affects the gene expression or linkage disequilibrium of other functional gene polymorphisms, which enhances the risk of RCC. These hypotheses require further investigation. We did find that *NLRP3* rs12143966 and *NLRP3* rs10925025, or and *NLRP3* rs10925026 had a significant additive and multiplicative interactions on the risk of RCC. However, we cannot explain these results at this time. Perhaps in addition to the total urinary arsenic concentration and the *NLRP3* rs1539019 genotype interaction, gene-gene interactions may also increase the risk of RCC.

This study had some limitations. This study was a case-control study design, therefore, we cannot rule out that the association between the environmental factors and RCC may be caused by RCC rather than the cause of RCC. A total of 15 SNPs of the *NLRP3* gene were examined in this study. However, the correlation between the *NLRP3* gene polymorphisms and *NLRP3* expression could not be assessed. Therefore, the interpretation of the results should be conservative. Because the sample size was not large; the interpretation of the significance of the findings should be limited. This study showed the joint effect of total urinary arsenic concentration and the *NLRP3* genotypes on the risk of developing RCC. However, we did not analyze the interaction between environmental arsenic exposure and the *NLRP3* genotype.

## Conclusions

This study is the first to identify significant multiplicative and additive interactions between high total urinary arsenic levels, the *NLRP3* rs1539019 C allele, and an increased risk of RCC. Furthermore, the *NLRP3* Five Tag-SNPs C-A-A-A-A haplotype had a higher OR for RCC than other haplotypes. Our data demonstrate that *NLRP3* gene polymorphisms could alter the correlation between total urinary arsenic concentrations and RCC, even in people with low arsenic exposure. In addition, the multivariate analyses showed that high total urinary arsenic concentration and the *NLRP3* rs1539019 C allele may indicate a higher OR for RCC. However, these results should be verified using a larger dataset.

## Supplementary information

Supplementary information
