# Supplementary material for: Polymorphism of nucleotide binding domain-like receptor protein 3 (NLRP3) increases susceptibility of total urinary arsenic to renal cell carcinoma
Source: Sci Rep. 2020 Apr 20;10:6640. doi: 10.1038/s41598-020-63469-8 (PMC7171170; doi:10.1038/s41598-020-63469-8)
Supplement: Supplementary file 1 — Supplementary information [file 41598_2020_63469_MOESM1_ESM.doc]

**Polymorphism of nucleotide binding domain-like receptor protein 3 (NLRP3) increases susceptibility of total urinary arsenic to renal cell carcinoma**

Chi-Jung Chung, PhD ^1,2^, Bo-Ying Bao, PhD ^3,4,5^, Ying-Chin Lin, MD ^6,7,8^, Ya-Li Huang, MSc ^9^, Horng-Sheng Shiue, MD, PhD ^10^, Pui-Lam Ao, MSc ^11^, Yeong-Shiau Pu, MD, PhD ^12^, Chao-Yuan Huang, MD, PhD ^12*^, Yu-Mei Hsueh, PhD ^6,9*^

^1^ Department of Public Health, College of Public Health, China Medical University, Taichung, Taiwan.

^2^ Department of Medical Research, China Medical University and Hospital, Taichung, Taiwan.

^3^ Department of Pharmacy, College of Pharmacy, China Medical University, Taichung, Taiwan.

^4^ Sex Hormone Research Center, China Medical University Hospital, Taichung, Taiwan.

^5^ Department of Nursing, Asia University, Taichung, Taiwan.

^6^ Department of Family Medicine, Wan Fang Hospital, Taipei Medical University, New Taipei City, Taiwan.

^7^ Department of Family Medicine, School of Medicine, College of Medicine, Taipei Medical University, Taipei, Taiwan.

^8^ Department of Health Examination, Wan Fang Hospital, Taipei Medical University, Taipei, Taiwan.

^9^ Department of Public Health, School of Medicine, College of Medicine, Taipei Medical University, Taipei, Taiwan

^10^ Department of Chinese Medicine, College of Medicine, Chang Gung University Taoyuan, Taiwan.

^11^ School of Public Health, College of Public Health, Taipei Medical University, Taipei, Taiwan

^12^ Department of Urology, National Taiwan University Hospital, College of Medicine National Taiwan University, Taipei, Taiwan

Supplemental Tables

Table S1 Pretreatment of urine samples, method of measurement, validity, and reliability of urinary arsenic species*

| Pretreatment of samples | Method of measurement | Variables | Detection limit (μg/L) | Recovery rate^ | SRM 2670^#^, Inorganic arsenic (mean ± SD) | CV% |
| --- | --- | --- | --- | --- | --- | --- |
| Urine samples were thawed at RT, sonicated, and filtered through a Sep-Pak C_18_ column. | HPLC-HG-AAS with Phenomenex C_18_ columns | As^III^ | 0.02 | 93.8%–102.2% | Certificate value  480 ± 100 μg/L  Measured value  507 ± 17 μg/L (n = 4) | < 10% |
|  |  | As^V^ | 0.10 |  |  |  |
|  |  | MMA^V^ | 0.07 |  |  |  |
|  |  | DMA^V^ | 0.06 |  |  |  |

^*^As^V^, arsenate; As^III^, arsenite; CV, coefficient of variation; DMA^V^, Dimethylarsinic acid; HG-AAS, hydride generator-atomic absorption spectrometry; HPLC, high-performance liquid chromatography; MMA^V^, monomethylarsonic acid; RT, room temperature

^The recovery rates of the four arsenic species were calculated as follows: [(sample-spiked standard solution concentration) – sample concentration] / standard solution concentration × 100.

^#^The standard reference material (SRM 2670) was obtained from the National Institute of Standards and Technology, Gaithersburg, MD, USA

Table S2 Comparisons of cumulative cigarette smoking among various genotypes of NLRP3

| Genotypes and haplotypes  of *NLRP3* | Total | *p* | Controls | *p* | RCC cases | *p* |
| --- | --- | --- | --- | --- | --- | --- |
| SNP1: rs4925654 | |  |  |  |  |  |
| GG | 9.27 ± 0.70 | 0.3112 | 8.07 ± 0.80 | 0.2966 | 11.76 ± 1.36 | 0.3131 |
| GA | 9.39 ± 1.23 |  | 9.71 ± 1.54 |  | 8.79 ± 2.05 |  |
| AA | 3.32 ± 1.96 |  | 2.80 ± 2.40 |  | 4.17 ± 3.53 |  |
| SNP2: rs4925650 | |  |  |  |  |  |
| GG | 8.11 ± 1.07 | 0.3831 | 6.33 ± 1.13 | 0.0572 | 11.86 ± 2.30 | 0.7563 |
| GA | 9.94 ± 0.87 |  | 9.96 ± 1.05 |  | 9.91 ± 1.57 |  |
| AA | 8.64 ± 1.22 |  | 7.17 ± 1.48 |  | 11.01 ± 2.09 |  |
| SNP3: rs12239046 | |  |  |  |  |  |
| CC | 8.56 ± 1.07 | 0.6127 | 7.57 ± 1.28 | 0.5447 | 10.27 ± 1.88 | 0.9405 |
| CT | 9.10 ± 0.80 |  | 8.31 ± 0.91 |  | 10.81 ± 1.60 |  |
| TT | 10.28± 1.48 |  | 9.81 ± 1.80 |  | 11.39 ± 2.59 |  |
| SNP4: rs4925648 | |  |  |  |  |  |
| CC | 9.02 ± 0.82 | 0.3214 | 8.66 ± 1.02 | 0.1594 | 9.74 ± 0.35 | 0.5653 |
| CT | 9.87 ± 0.97 |  | 8.81 ± 1.03 |  | 12.00 ± 2.04 |  |
| TT | 6.21 ± 1.66 |  | 3.64 ± 1.41 |  | 12.75 ± 4.43 |  |
| SNP5: rs10925025 | |  |  |  |  |  |
| AA | 10.39 ± 1.49 | 0.5981 | 9.89 ± 1.81 | 0.5397 | 11.61 ± 2.63 | 0.9304 |
| AG | 9.12 ± 0.80 |  | 8.31 ± 0.91 |  | 10.83 ± 1.59 |  |
| GG | 8.62 ± 0.08 |  | 7.62 ± 1.30 |  | 10.35 ± 1.90 |  |
| SNP6: rs1539019 | |  |  |  |  |  |
| CC | 8.58 ± 1.06 | 0.5980 | 7.68 ± 1.28 | 0.5737 | 10.12 ± 1.86 | 0.8874 |
| CA | 9.11 ± 0.80 |  | 8.24 ± 0.91 |  | 10.98 ± 1.60 |  |
| AA | 10.35 ± 1.50 |  | 9.82 ± 1.83 |  | 11.61 ± 2.63 |  |
| SNP7: rs3806265 | |  |  |  |  |  |
| TT | 8.80 ± 1.07 | 0.3448 | 8.16 ± 1.34 | 0.1427 | 9.96 ± 1.77 | 0.8099 |
| TC | 9.92 ± 0.89 |  | 9.50 ± 1.07 |  | 10.74 ± 1.61 |  |
| CC | 7.72 ± 1.13 |  | 6.00 ± 1.09 |  | 12.09 ± 2.82 |  |
| SNP8: rs10925026 | |  |  |  |  |  |
| CC | 10.39 ± 1.49 | 0.5709 | 9.89 ± 1.81 | 0.5176 | 11.61 ± 2.63 | 0.9200 |
| CA | 9.03 ± 0.80 |  | 8.18 ± 0.91 |  | 10.83 ± 1.59 |  |
| AA | 8.56 ± 1.07 |  | 7.57 ± 1.28 |  | 10.27 ± 1.88 |  |
| SNP9: rs10157379 | |  |  |  |  |  |
| CC | 10.45 ± 1.50 | 0.5778 | 9.97 ± 1.83 | 0.5213 | 11.61 ± 2.63 | 0.9315 |
| CT | 9.00 ± 0.79 |  | 8.18 ± 0.90 |  | 10.76 ± 1.58 |  |
| TT | 8.65 ± 1.08 |  | 7.67 ± 1.30 |  | 10.35 ± 1.90 |  |
| SNP10: rs12143966 | |  |  |  |  |  |
| GG | 10.00 ± 1.20 | 0.7454 | 9.95 ± 1.45 | 0.4272 | 10.14 ± 2.15 | 0.9579 |
| GA | 8.90 ± 0.81 |  | 7.86 ± 0.91 |  | 10.94 ± 1.58 |  |
| AA | 8.97 ± 1.27 |  | 7.82 ± 1.54 |  | 10.99 ± 2.23 |  |
| SNP11: rs10754555 | |  |  |  |  |  |
| CC | 8.85 ± 0.97 | 0.5399 | 8.17 ± 1.23 | 0.2389 | 10.06 ± 1.54 | 0.7981 |
| CG | 9.78 ± 0.86 |  | 9.31 ± 0.96 |  | 10.85 ± 1.78 |  |
| GG | 7.98 ± 1.53 |  | 5.91 ± 1.66 |  | 12.24 ± 3.13 |  |
| SNP12: rs3806268 | |  |  |  |  |  |
| AA | 8.90 ± 1.06 | 0.3967 | 8.25 ± 1.34 | 0.1537 | 10.10 ± 1.76 | 0.7945 |
| AG | 9.84 ± 0.90 |  | 9.46 ± 1.08 |  | 10.59 ± 1.61 |  |
| GG | 7.77 ± 1.13 |  | 6.02 ± 1.09 |  | 12.30 ± 2.86 |  |
| SNP13: rs12048215 | |  |  |  |  |  |
| AA | 8.96 ± 0.88 | 0.4199 | 8.54 ± 1.11 | 0.1396 | 9.76 ± 1.42 | 0.5586 |
| AG | 9.82 ± 0.92 |  | 9.14 ± 1.03 |  | 11.25 ± 1.86 |  |
| GG | 7.19 ± 1.60 |  | 4.54 ± 1.40 |  | 13.77 ± 4.18 |  |

Table S3 Comparisons of urinary arsenic among various genotypes of NLRP3

| Genotype of *NLRP3* | Total | *p* | Control | *p* | RCC | *p* |
| --- | --- | --- | --- | --- | --- | --- |
| SNP1: rs4925654 | |  |  |  |  |  |
| GG | 20.47 ± 0.63 | 0.9777 | 18.70 ± 0.57 | 0.7529 | 24.10 ± 1.51 | 0.8385 |
| GA | 20.60 ± 0.94 |  | 19.57 ± 1.07 |  | 22.61 ± 1.83 |  |
| AA | 19.87 ± 3.20 |  | 18.76 ± 4.04 |  | 21.89 ± 5.52 |  |
| SNP2: rs4925650 | |  |  |  |  |  |
| GG | 18.74 ± 0.88 ^b^ | 0.0049 | 17.27 ± 0.91 | 0.1099 | 21.84 ± 1.91 | 0.0176 |
| GA | 20.21 ± 0.67 ^a^ |  | 19.53 ± 0.75 |  | 21.69 ± 1.39^a^ |  |
| AA | 23.48 ± 1.41 ^a,b^ |  | 19.71 ± 0.95 |  | 29.43 ± 3.21^a^ |  |
| SNP3: rs12239046 | |  |  |  |  |  |
| CC | 19.20 ± 0.82 | 0.1659 | 18.04 ± 0.78 | 0.2306 | 21.20 ± 1.79 | 0.1910 |
| CT | 21.37 ± 0.72 |  | 19.80 ± 0.76 |  | 24.72 ± 1.54 |  |
| TT | 20.83 ± 1.55 |  | 18.14 ± 1.21 |  | 27.08 ± 4.23 |  |
| SNP4: rs4925648 | |  |  |  |  |  |
| CC | 20.99 ± 0.72 | 0.3047 | 19.00 ± 0.63 | 0.0652 | 24.90 ± 1.73 | 0.4215 |
| CT | 20.26 ± 0.79 |  | 19.58 ± 0.95 |  | 21.63 ± 1.41 |  |
| TT | 17.85 ± 2.05 |  | 14.85 ± 1.18 |  | 24.80 ± 6.08 |  |
| SNP5: rs10925025 | |  |  |  |  |  |
| AA | 20.94 ± 1.56 | 0.1945 | 18.24 ± 1.22 | 0.3778 | 27.32 ± 4.30 | 0.1493 |
| AG | 21.21 ± 0.71 |  | 19.56 ± 0.73 |  | 24.67 ± 1.53 |  |
| GG | 19.18 ± 0.83 |  | 18.15 ± 0.79 |  | 20.95 ± 1.79 |  |
| SNP6: rs1539019 | |  |  |  |  |  |
| CC | 19.22 ± 0.82 | 0.1598 | 18.10 ± 0.78 | 0.2065 | 21.13 ± 1.77 | 0.1621 |
| CA | 21.42 ± 0.72 |  | 19.84 ± 0.76 |  | 24.77 ± 1.54 |  |
| AA | 20.77 ± 1.57 |  | 17.97 ± 1.20 |  | 27.32 ± 4.30 |  |
| SNP7: rs3806265 | |  |  |  |  |  |
| TT | 22.43 ± 1.14 ^b^ | 0.0213 | 19.58 ± 0.92 | 0.0732 | 27.52 ± 2.66 | 0.0876 |
| TC | 20.26 ± 0.67 |  | 19.47 ± 0.75 |  | 21.81 ± 1.32 |  |
| CC | 18.35 ± 1.04 ^b^ |  | 16.75 ± 0.95 |  | 22.30 ± 2.71 |  |
| SNP8: rs10925026 | |  |  |  |  |  |
| CC | 20.94 ± 1.56 | 0.1700 | 18.24 ± 1.22 | 0.2649 | 27.32 ± 4.30 | 0.1784 |
| CA | 21.34 ± 0.72 |  | 19.74 ± 0.76 |  | 24.67 ± 1.53 |  |
| AA | 19.20 ± 0.82 |  | 18.04 ± 0.78 |  | 21.20 ± 1.79 |  |
| SNP9: rs10157379 | |  |  |  |  |  |
| CC | 21.02 ± 1.57 | 0.1693 | 18.33 ± 1.23 | 0.2524 | 27.32 ± 4.30 | 0.1889 |
| CT | 21.33 ± 0.71 |  | 19.78 ± 0.75 |  | 24.61 ± 1.52 |  |
| TT | 19.20 ± 0.83 |  | 18.02 ± 0.79 |  | 21.25 ± 1.80 |  |
| SNP10: rs12143966 | |  |  |  |  |  |
| GG | 21.39 ± 1.26 | 0.1825 | 18.67 ± 1.01 | 0.2007 | 28.19 ± 3.50 | 0.0997 |
| GA | 20.83 ± 0.70 |  | 19.83 ± 0.76 |  | 22.80 ± 1.44 |  |
| AA | 18.93 ± 0.94 |  | 17.68 ± 0.90 |  | 21.10 ± 2.05 |  |
| SNP11: rs10754555 | |  |  |  |  |  |
| CC | 21.47 ± 0.93 | 0.2847 | 19.11 ± 0.75 | 0.5686 | 25.65 ± 2.19 | 0.3846 |
| CG | 20.15 ± 0.70 |  | 19.16 ± 0.77 |  | 22.34 ± 1.43 |  |
| GG | 19.22 ± 1.27 |  | 17.69 ± 1.30 |  | 22.29 ± 2.77 |  |
| SNP12: rs3806268 | |  |  |  |  |  |
| AA | 22.40 ± 1.13 ^b^ | 0.0307 | 19.56 ± 0.91 | 0.1534 | 27.51 ± 2.63 | 0.0862 |
| AG | 20.16 ± 0.66 |  | 19.34 ± 0.74 |  | 21.76 ± 1.32 |  |
| GG | 18.59 ± 1.07 ^b^ |  | 17.09 ± 1.01 |  | 22.38 ± 2.75 |  |
| SNP13: rs12048215 | |  |  |  |  |  |
| AA | 20.78 ± 0.79 | 0.3890 | 18.66 ± 0.67 | 0.1373 | 24.77 ± 1.87 | 0.6735 |
| AG | 20.73 ± 0.76 |  | 19.82 ± 0.87 |  | 22.61 ± 1.49 |  |
| GG | 18.43 ± 1.56 |  | 16.54 ± 1.26 |  | 22.88 ± 4.25 |  |

^a^ or ^b^ p<0.05 calculating through scheffe’s tests

| Table S4 The interactions of inflammasome gene polymorphisms on RCC risk | | | | | |
| --- | --- | --- | --- | --- | --- |
| Polymorphisms of *NLRP3* | |  | Multivariate ORs (95% CI)^a^ | P_interaction_ | S index |
| rs10925025 | rs1539019 |  |  |  |  |
| AA or AG | CC or CA | 161/337 | 1.00 | -- |  |
| AA or AG | AA | 53/124 | 0.84 (0.56-1.26) |  |  |
| GG | CC or CA | 135/232 | 1.21 (0.89-1.65) |  |  |
| GG | AA | 0/0 |  |  |  |
|  |  |  |  |  |  |
| rs10925025 | rs10925026 |  |  |  |  |
| AA or AG | CC | 214/461 | 1.00 | -- |  |
| AA or AG | CA or AA | 0/0 |  |  |  |
| GG | CC | 0/0 |  |  |  |
| GG | CA or AA | 135/232 | 1.27 (0.95-1.70) |  |  |
|  |  |  |  |  |  |
| rs10925025 | rs12143966 |  |  |  |  |
| AA or AG | GG | 65/173 | 1.00^*^ | 0.0245 | 0.07 (0.01-0.54) |
| AA or AG | AG or AA | 144/280 | 1.53 (1.04-2.25) |  |  |
| GG | GG | 5/2 | 8.98 (1.52-53.02) |  |  |
| GG | AG or AA | 130/230 | 1.63 (1.10-2.41) |  |  |
|  |  |  |  |  |  |
| rs1539019 | rs10925026 |  |  |  |  |
| CC or CA | CC | 161/337 | 1.00 | -- |  |
| CC or CA | CA or AA | 136/236 | 1.17 (0.86-1.59) |  |  |
| AA | CC | 53/124 | 0.83 (0.55-1.24) |  |  |
| AA | CA or AA | 0/0 |  |  |  |
|  |  |  |  |  |  |
| rs1539019 | rs12143966 |  |  |  |  |
| CC or CA | GG | 17/53 | 1.00 | 0.9807 |  |
| CC or CA | AG or AA | 274/513 | 1.21 (0.88-1.64) |  |  |
| AA | GG | 53/122 | 0.84 (0.56-1.27) |  |  |
| AA | AG or AA | 0/1 |  |  |  |
|  |  |  |  |  |  |
| rs10925026 | rs12143966 |  |  |  |  |
| CC | GG | 65/172 | 1.00^+^ | 0.0240 | 0.07 (0.01-0.52) |
| CC | AG or AA | 144/281 | 1.51 (1.03-2.21) |  |  |
| CA or AA | GG | 5/2 | 8.89 (1.51-52.25) |  |  |
| CA or AA | AG or AA | 130/234 | 1.59 (1.08-2.35) |  |  |
| **^+^**trend 0.05< p-value < 0.1. ^a^ Model was adjusted by age, sex, cumulative cigarette smoking, alcohol consumption, total urinary arsenic levels, diabetes, and hypertension. | | | | | |
